# Case Report: Elastography-negative acute fatty liver of pregnancy diagnosed by histopathology and electron microscopy

**DOI:** 10.3389/fmed.2026.1820682

**Published:** 2026-06-15

**Authors:** Takuto Nosaka, Arisa Saito, Yu Akazawa, Kazuto Takahashi, Tatsushi Naito, Masahiro Ohtani, Yoshiaki Imamura, Sho Kudo, Makoto Orisaka, Yoshio Yoshida, Yasunari Nakamoto

**Affiliations:** 1 Second Department of Internal Medicine, Faculty of Medical Sciences, University of Fukui, Fukui, Japan; 2 Division of Diagnostic Pathology/Surgical Pathology, University of Fukui Hospital, Fukui, Japan; 3 Department of Obstetrics and Gynecology, Faculty of Medical Sciences, University of Fukui, Fukui, Japan

**Keywords:** acute fatty liver of pregnancy, controlled attenuation parameter, electron microscopy, microvesicular steatosis, transient elastography

## Abstract

Acute fatty liver of pregnancy (AFLP) is a rare pregnancy-related liver disorder that can rapidly progress to multiorgan failure, including disseminated intravascular coagulation (DIC) and renal dysfunction. It is potentially fatal if not promptly recognized and treated; however, the diagnostic role of imaging modalities remains poorly defined. We report a case of a 29-year-old primigravida who presented with progressive jaundice, liver dysfunction, coagulopathy, and renal impairment at 33 weeks of gestation. Abdominal computed tomography (CT) demonstrated decreased hepatic attenuation, with a CT value of 23.0 Hounsfield units, suggestive of fatty deposition. Based on fulfillment of 7 of the 14 Swansea criteria and the presence of fetal distress, an emergency cesarean section was performed. On postoperative day 18, the controlled attenuation parameter (CAP) obtained by vibration-controlled transient elastography indicated a steatosis grade of S0, with a CAP value of 196 dB/m, whereas ultrasound-guided liver biopsy revealed microvesicular steatosis on histological examination, confirmed by adipophilin immunostaining. Electron microscopy further demonstrated numerous microvesicular lipid droplets and dilatation of the rough endoplasmic reticulum, leading to a definitive diagnosis of AFLP. This case suggests that CAP may underestimate hepatic fat accumulation when steatosis is predominantly microvesicular, as in AFLP, and that detailed pathological and ultrastructural evaluation may be valuable for diagnostic confirmation and improved understanding of disease pathophysiology.

## 1 Introduction

Acute fatty liver of pregnancy (AFLP) is a rare but life-threatening pregnancy-related liver disorder that typically develops in late pregnancy or the early postpartum period (1, 2). The incidence is reported to be approximately 0.005–0.014%, and the condition can rapidly progress to multiorgan failure, including acute liver failure, disseminated intravascular coagulation (DIC), and renal dysfunction. Reported maternal mortality rates range from 16.5 to 26.7%, and perinatal mortality rates range from 10 to 200 per 1,000 births (3, 4). Early diagnosis and prompt termination of pregnancy are essential to improve maternal and fetal outcomes (1).

At present, no definitive diagnostic criteria for AFLP have been established. Histological evaluation has been reported to be useful for establishing a definitive diagnosis (5). Although imaging modalities such as ultrasonography, computed tomography (CT), and magnetic resonance imaging (MRI) of the liver may help support the diagnosis of AFLP, their diagnostic role remains unclear (4). As a noninvasive assessment of hepatic steatosis, the controlled attenuation parameter (CAP) obtained by FibroScan based on vibration-controlled transient elastography has been extensively validated in metabolic dysfunction–associated steatotic liver disease (MASLD) and demonstrates good agreement with liver biopsy findings (6). However, it is also known that in certain pathological conditions, CAP does not accurately reflect the degree of hepatic fat accumulation (7).

We report a case of AFLP complicated by DIC and renal dysfunction, in which both the patient and the fetus survived following prompt diagnosis and emergency delivery. Although hepatic fat deposition was detected on CT, ultrasonographic elastography failed to accurately evaluate hepatic steatosis. A definitive diagnosis of AFLP was ultimately established based on a detailed pathological evaluation of liver biopsy specimens, including immunohistochemical staining and electron microscopy.

## 2 Case description

**Figure 1:**
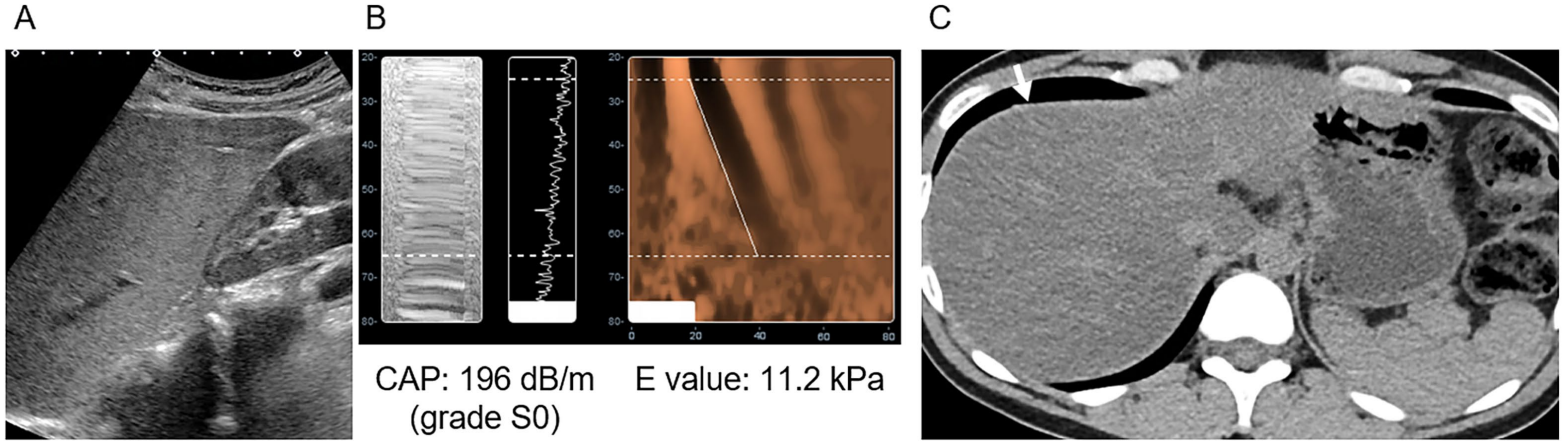
Imaging findings at the onset of acute fatty liver of pregnancy. **(A)** Abdominal ultrasonography showing mildly increased hepatorenal contrast. **(B)** Representative images obtained by vibration-controlled transient elastography (FibroScan®), showing the controlled attenuation parameter (CAP) and liver stiffness (E value). **(C)** Non-contrast abdominal computed tomography demonstrating diffusely decreased hepatic parenchymal attenuation, with a CT value of 23.0 Hounsfield units (HU; white arrow).

A 29-year-old primigravida conceived naturally and had an uneventful clinical course until 32 weeks of gestation. The patient had no relevant medical history, including liver disease, diabetes mellitus, dyslipidemia, or inherited metabolic disorders. She had no history of alcohol consumption and no family history suggestive of liver disease or fatty acid oxidation disorders. Her pre-pregnancy BMI was 24.1 kg/m^2^. At 32 weeks and 4 days of gestation, elevated hepatobiliary enzyme levels were detected on routine blood testing, and she was emergently admitted to the referring hospital. Despite hospitalization, hepatobiliary enzyme levels continued to increase, and at 33 weeks and 6 days of gestation (Day 0), she developed worsening uterine contractions and jaundice. She was subsequently transferred emergently to the Department of Obstetrics and Gynecology at our hospital for further evaluation and management and was referred to our department. On admission, her vital signs were as follows: body temperature, 35.8 °C; blood pressure, 137/87 mmHg. Physical examination revealed no abdominal tenderness, but marked jaundice of the skin and sclera. On admission, laboratory investigations revealed elevated hepatobiliary enzyme levels, along with coagulopathy, renal dysfunction, and hypoglycemia (Table 1). Although the white blood cell count was elevated to 17,600/μL on admission, the CRP level was low and the patient had no fever or obvious clinical focus of infection. Therefore, leukocytosis was interpreted in the context of late pregnancy and acute physiological stress associated with AFLP and fetal distress, rather than as evidence of bacterial infection. Abdominal ultrasonography demonstrated only a mild increase in the hepatorenal contrast (Figure 1A). In contrast, abdominal CT revealed decreased hepatic parenchymal attenuation, with a CT value of 23.0 Hounsfield units (Figure 1C). The patient fulfilled 7 of the 14 Swansea criteria, including vomiting, hyperbilirubinemia, hypoglycemia, leukocytosis, liver dysfunction, coagulopathy, and imaging findings suggestive of fatty liver. Based on these findings, she was clinically diagnosed with AFLP complicated by DIC and renal dysfunction. In addition, fetal heart rate monitoring revealed reduced baseline variability and late decelerations, indicating fetal distress. After administration of fresh frozen plasma and antithrombin III concentrate, an emergency cesarean section was performed, resulting in the delivery of a male infant. Blood tests confirmed the absence of viral hepatitis infection and autoimmune liver disease.

**Table 1:** Laboratory examinations.

Parameter	Value	Unit
Complete blood count		
WBCs	17,600	/μL
RBCs	4.41 × 10^6^	/μL
Hemoglobin	13.6	g/dL
Hematocrit	42.3	%
Platelets	81 × 103	/μL
Blood coagulation test		
PT	20.1	sec
PT-INR	1.65	
APTT	46.3	sec
Fibrinogen	<50	mg/dL
FDP	41	μg/mL
D-dimer	13.6	μg/mL
AT-III	87	%
Serum chemistry		
AST	68	U/L
ALT	67	U/L
LDH	360	U/L
ALP	633	U/L
γGTP	336	U/L
Total bilirubin	11.9	mg/dL
Direct bilirubin	8.3	mg/dL
Total protein	5.3	g/dL
Albumin	3.1	g/dL
BUN	16	mg/dL
Creatinine	1.48	mg/dL
UA	5.5	mg/dL
Glucose	54	mg/dL
NH3	37	μmol/L
Serum immunological test		
CRP	0.24	mg/dL
IgG	993	mg/dL
IgM	115	mg/dL
IgA	177	mg/dL
ANA	<1:40	
AMA-M2	(−)	
Viral markers		
Anti-HAV IgG	4.95	S/CO
IgM anti-HAV	(−)	
HBsAg	(−)	
HBV DNA	(−)	
Anti-HCV Ab	(−)	
HCV RNA	(−)	
IgA anti-HEV	(−)	
IgM anti-CMV	(−)	
IgG anti-CMV	197.0	AU/mL
IgM anti-HSV	(−)	
IgG anti-HSV	12.9	(<2.0)
EBV VCA IgM	(−)	
EBV VCA IgG	80	(<10)
EBNA	20	(<10)
Thyroid function		
Free T3	1.89	pg/mL
Free T4	0.85	ng/mL
TSH	2.495	μIU/mL

WBCs, white blood cells; RBCs, red blood cells; PT, prothrombin time; PT-INR, prothrombin time–international normalized ratio; APTT, activated partial thromboplastin time; Fibrinogen, fibrinogen; FDP, fibrinogen/fibrin degradation products; AT-III, antithrombin III; AST, aspartate aminotransferase; ALT, alanine aminotransferase; LDH, lactate dehydrogenase; ALP, alkaline phosphatase; γGTP, gamma-glutamyl transpeptidase; BUN, blood urea nitrogen; UA, uric acid; CRP, C-reactive protein; ANA, antinuclear antibody; AMA-M2, anti-mitochondrial M2 antibody; Ig, immunoglobulin; HAV, hepatitis A virus; HEV, hepatitis E virus; HBsAg, hepatitis B virus surface antigen; HCV Ab, anti–hepatitis C virus antibody; CMV, cytomegalovirus; EBV, Epstein–Barr virus; VCA, viral capsid antigen; EBNA, Epstein–Barr virus nuclear antigen; TSH, thyroid-stimulating hormone.

**Figure 2:**
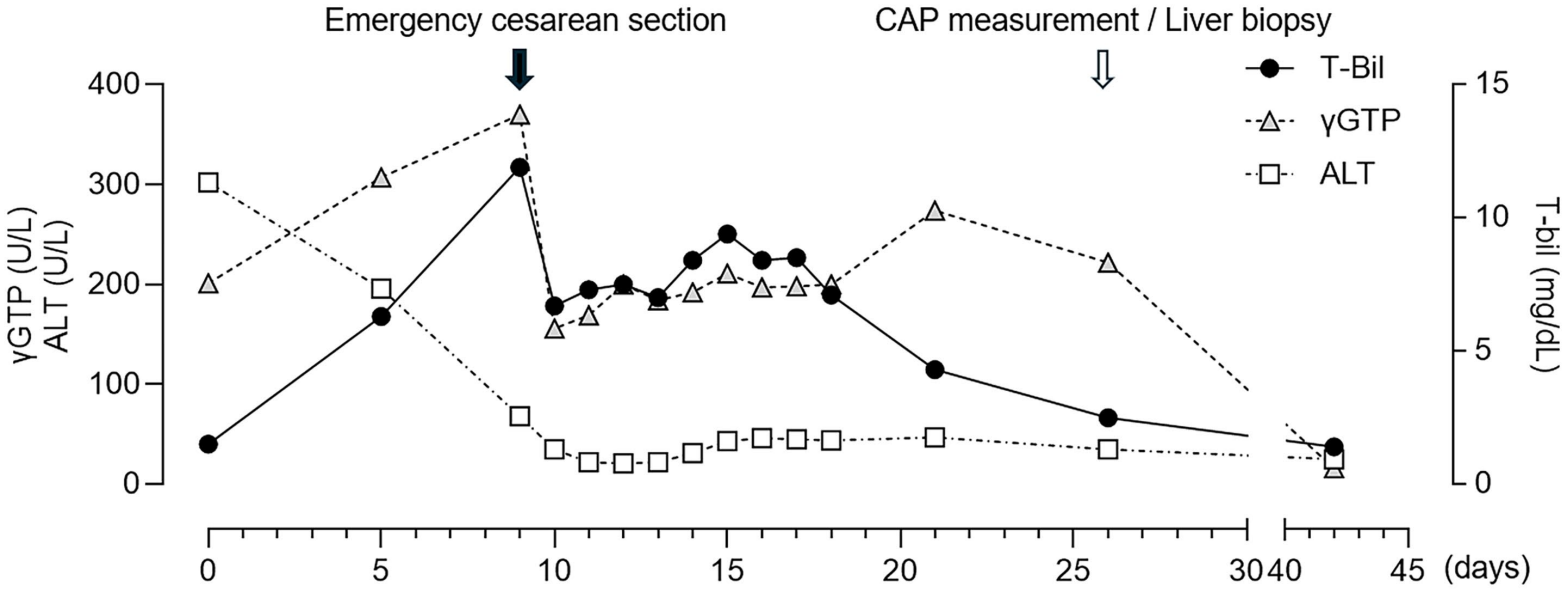
Clinical course of the patient. The clinical course from admission through delivery and the postpartum period is shown. Changes in laboratory findings and major clinical events are presented chronologically.

To evaluate hepatic steatosis and the underlying pathology, vibration-controlled transient elastography using FibroScan and ultrasound-guided liver biopsy were performed on postoperative day 18 (Figure 2). For FibroScan, the M probe was used according to the patient’s body habitus. A total of 10 valid measurements were obtained, with a success rate of 100%. The median CAP value was 196 dB/m, with an IQR of 23 dB/m. The median liver stiffness measurement was 11.2 kPa, with an IQR/median of 9%. Under these acquisition conditions, the CAP value corresponded to steatosis grade S0 (Figure 1B).

**Figure 3:**
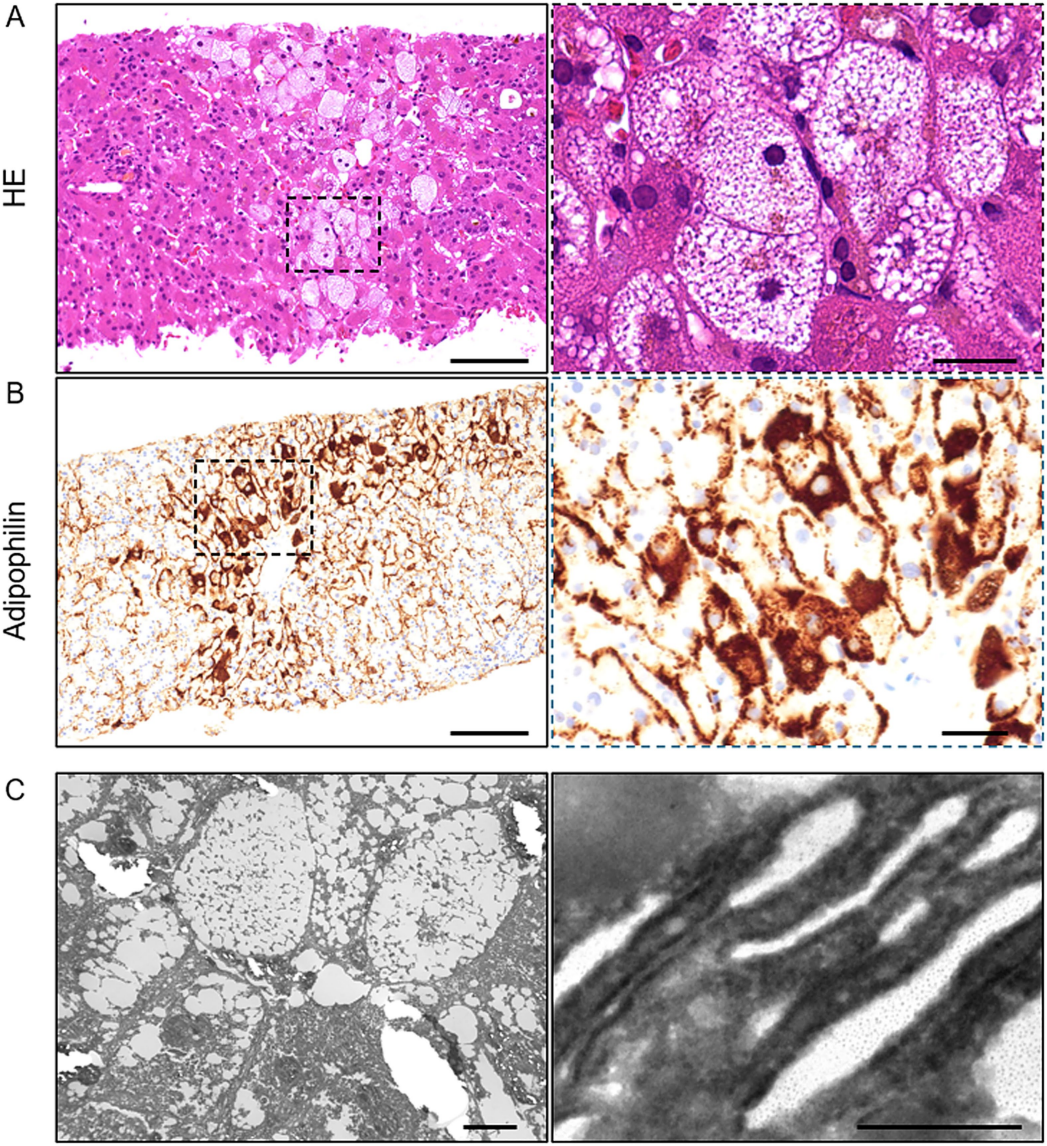
Histopathological and ultrastructural findings of the liver biopsy specimen. **(A)** Hematoxylin and eosin (HE)–stained section of the liver biopsy specimen showing numerous microvesicular lipid droplets within hepatocytes. Scale bars: left panel, 100 μm; right panel, 20 μm. **(B)** Immunohistochemical staining for adipophilin demonstrating positive staining of intracellular lipid droplets in hepatocytes. Scale bars: left panel, 100 μm; right panel, 20 μm. **(C)** Electron microscopic findings showing abundant microvesicular lipid droplets within the hepatocyte cytoplasm (left panel, Bar, 10 μm) and dilatation of the rough endoplasmic reticulum (right panel, Bar, 600 nm).

Histological examination of the liver biopsy specimens revealed numerous microvesicular lipid droplets in hepatocytes predominantly around the central veins, and immunohistochemical staining for adipophilin confirmed the presence of microvesicular steatosis (Figures 3A,B). Electron microscopy demonstrated abundant microvesicular lipid droplets within the hepatocyte cytoplasm, along with dilatation of the rough endoplasmic reticulum (Figure 3C). Based on these histopathological findings, a definitive diagnosis of AFLP was established. HELLP syndrome, acute viral hepatitis, and autoimmune liver disease were considered as important differential diagnoses. HELLP syndrome was clinically relevant because the patient developed thrombocytopenia and liver dysfunction in late pregnancy. However, the presence of jaundice, hypoglycemia, renal impairment, coagulopathy, DIC, and fulfillment of multiple Swansea criteria favored AFLP. Acute viral hepatitis was considered unlikely because serological and molecular tests for hepatitis A, B, C, and E viruses were negative, and the clinical course was not suggestive of acute viral infection. Autoimmune liver disease was also considered unlikely based on negative autoantibodies and the absence of marked hypergammaglobulinemia. The subsequent demonstration of microvesicular steatosis by liver biopsy further supported the diagnosis of AFLP. Hepatobiliary enzyme levels continued to decrease and showed further improvement by Day 42, with complete normalization confirmed by Day 82.

## 3 Discussion

We experienced a case of AFLP complicated by DIC and renal dysfunction, in which both the patient and the fetus were successfully rescued through prompt clinical diagnosis and emergency delivery. Although hepatic fat deposition was detected on CT, ultrasonographic elastography failed to accurately evaluate hepatic steatosis. A definitive diagnosis of AFLP was ultimately established based on detailed pathological evaluation of liver biopsy specimens using immunohistochemical staining and electron microscopy.

Reported risk factors for AFLP include multiple gestation, male fetus, coexisting liver disease, fetal disorders of fatty acid oxidation—particularly long-chain 3-hydroxyacyl-CoA dehydrogenase (LCHAD) deficiency—and a prior history of acute fatty liver of pregnancy (1, 4). The pathophysiology of AFLP is characterized by microvesicular steatosis resulting from impaired mitochondrial *β*-oxidation of fatty acids (8). A widely accepted mechanism is that fetal fatty acid oxidation defects, such as LCHAD deficiency, allow intermediate metabolites to enter the maternal circulation, thereby inducing maternal hepatic fatty acid metabolic dysfunction and leading to diffuse accumulation of microvesicular lipid droplets within hepatocytes (9). In the present case, routine neonatal screening after birth did not reveal any findings suggestive of fatty acid oxidation disorders. However, because detailed genetic testing for fatty acid oxidation disorders, including LCHAD deficiency, was not performed, fetal fatty acid oxidation disorders could not be completely excluded. This represents a limitation of the etiological evaluation in the present case. Because fetal fatty acid oxidation defects may contribute to the development of maternal liver injury in AFLP, neonatal screening, metabolic assessment, and, when appropriate, genetic testing should be considered.

In the present case, CT demonstrated findings suggestive of diffuse hepatic fat deposition, whereas the CAP measured by FibroScan indicated S0 and failed to accurately reflect the degree of hepatic steatosis. In general, liver imaging modalities, including ultrasonography, CT, and MRI, may aid in the diagnosis of AFLP; however, their diagnostic role remains unclear (4). Although CT can detect hepatic hypoattenuation reflecting fatty infiltration and has been reported to be useful for diagnosing AFLP (10), typical imaging findings are present in only approximately 50% of cases (4). Ultrasonography, in contrast, has been reported to detect fatty liver findings in only about 25% of cases in a prospective study (2, 4).

A possible explanation for the discrepancy between CT and CAP findings in the present case is the difference in acoustic properties between microvesicular and macrovesicular steatosis. CAP estimates hepatic steatosis by measuring ultrasound attenuation through the liver parenchyma, and its diagnostic performance has been validated mainly in studies of macrovesicular steatosis in MASLD (11). In macrovesicular steatosis, large intracellular lipid vacuoles displace the nucleus and cytoplasmic organelles, thereby producing relatively prominent changes in tissue architecture, acoustic impedance, scattering, and attenuation. In contrast, microvesicular steatosis, which is characteristic of AFLP, consists of numerous small lipid droplets diffusely distributed within the hepatocyte cytoplasm (5, 8). These small lipid droplets may produce less pronounced changes in acoustic impedance and ultrasound attenuation within the measurement volume of CAP. Differences in droplet size, distribution pattern, and effects on tissue architecture may therefore explain why CAP indicated S0 in the present case despite histological evidence of microvesicular steatosis.

However, this interpretation remains hypothetical because the diagnostic performance of CAP for microvesicular steatosis has not been systematically investigated. Further studies comparing CAP, CT, MRI, and histopathological findings in diseases predominantly characterized by microvesicular steatosis, such as AFLP and drug-induced mitochondrial hepatopathy, are warranted.

In this case, a liver biopsy was performed, and detailed pathological evaluation using immunohistochemical staining and electron microscopy revealed numerous microvesicular lipid droplets scattered throughout the hepatocyte cytoplasm, along with dilatation of the rough endoplasmic reticulum. Microscopically, acute fatty liver of pregnancy has been reported to show microvesicular steatosis, intrahepatic cholestasis, and mild lobular and portal inflammation (12). Characteristic electron microscopic findings in AFLP include microvesicular lipid droplets and mitochondrial abnormalities, such as swelling, pleomorphism, disruption of cristae, and paracrystalline inclusions (13). Consistent with these previous reports, immunohistochemical staining and electron microscopy in the present case demonstrated microvesicular lipid deposition characteristic of AFLP.

The present case report has several limitations. First, this is a single case report, and no generalizable conclusion can be drawn regarding the diagnostic performance of CAP in pregnant women or in patients with AFLP as a whole. Second, CAP has been validated primarily for the assessment of macrovesicular steatosis in MASLD, whereas its sensitivity and diagnostic accuracy for microvesicular steatosis, which is characteristic of AFLP, have not been sufficiently established. Third, pregnancy-related hemodynamic changes, cholestasis, hepatocellular injury, and hepatic edema may also have affected the ultrasonographic assessment. Therefore, this case should not be interpreted as proof of the limitations of CAP in AFLP, but rather as a hypothesis-generating case suggesting that CAP may underestimate hepatic fat accumulation in conditions predominantly characterized by microvesicular steatosis.

## 4 Conclusion

In the present case, hepatic fat deposition associated with AFLP was suggested by CT and confirmed as microvesicular steatosis by liver biopsy and electron microscopy, whereas CAP obtained by ultrasonographic elastography did not indicate significant hepatic steatosis. This discrepancy suggests that CAP may underestimate hepatic fat accumulation when steatosis is predominantly microvesicular, as in AFLP. Detailed pathological assessment, including electron microscopic evaluation, was useful for diagnostic confirmation and may contribute to a better understanding of the pathophysiology of AFLP. However, because this is a single case report, further accumulation and validation of cases are needed to clarify the diagnostic performance and limitations of CAP in AFLP.

## 5 Patient perspective

The patient reported anxiety when she was informed during pregnancy that she had jaundice and liver dysfunction and that emergency cesarean section was required for maternal and fetal safety. She was concerned about the potential effects of her liver dysfunction on the newborn and the severity of her disease. After delivery, her general condition and liver function gradually improved, and the diagnosis of AFLP was confirmed by liver biopsy. After receiving an explanation of the disease, she understood that AFLP is a rare but potentially life-threatening pregnancy-related liver disorder. She expressed relief that both she and her infant recovered without major complications.

## Data Availability

The original contributions presented in the study are included in the article/supplementary material, further inquiries can be directed to the corresponding authors.
